# Treatment-resistant or difficult-to-treat depression: a consensus on the pharmacotherapy challenges and considerations for the health care system in Greece

**DOI:** 10.3389/fpsyt.2025.1561821

**Published:** 2025-04-28

**Authors:** Kyriakos Souliotis, Christina Golna, Myrto Samara, Eva-Maria Tsapakis, Vasilios P. Bozikas, Thomas N. Hyphantis, Nikolaos Smyrnis, Nikos Stefanis

**Affiliations:** ^1^ Department of Social and Education Policy, University of Peloponnese, Corinth, Greece; ^2^ Research Department, The Health Policy Institute, Maroussi, Attica, Greece; ^3^ Department of Psychiatry, Faculty of Medicine, University of Thessaly, Larisa, Greece; ^4^ 3rd Department of Academic Psychiatry, Aristotle University of Thessaloniki, Thessaloniki, Greece; ^5^ 2nd Department of Psychiatry, School of Medicine, Psychiatric Hospital of Thessaloniki, Aristotle University of Thessaloniki, Thessaloniki, Greece; ^6^ Department of Psychiatry, Faculty of Medicine, School of Health Sciences, University of Ioannina, Ioannina, Greece; ^7^ 2nd Psychiatry Department, National and Kapodistrian University of Athens, Medical School, University General Hospital “ATTIKON“, Athens, Greece; ^8^ 1st Psychiatry Department, National and Kapodistrian University of Athens, “EGINITION” Hospital, Athens, Greece

**Keywords:** treatment resistant depression (TRD), difficult to treat depression (DTD), depression, integrated healthcare delivery, mental health policy

## Abstract

**Introduction:**

Globally, there is limited scientific consensus on the definition of Treatment Resistant Depression (TRD) or Difficult to Treat Depression (DTD) and even greater challenges are being reported with its management. In Greece, the last available guidelines on depression from 2015 make no reference to TRD/DTD management. This study aims to inform the definition of TRD or DTD and propose a pathway for its integrated management in the context of the Greek National Health System (NHS).

**Methods:**

Individual interviews with clinical experts based on a structured interview guide were conducted in November 2022 to explore consensus on the definition, key challenges, and prospects for the management of TRD/DTD in Greece. Results were combined in a manuscript that was circulated amongst authors for comments and sign off.

**Results:**

Participants preferred the use of the DTD term over TRD, though noted that using the term TRD may be more amendable to wider scientific audiences. They also agreed on the need to set bold treatment goals and assess optimal treatment dose, duration, and adherence, in the context of shared decision making, prior to confirming a diagnosis as TRD/DTD and proposing a treatment strategy. Integration of patient management with use of mobile mental health units, Mental Health Centers and tertiary Centers of Excellence would promote patient centricity, accessibility, affordability as well as help develop an evidence basis for the further customization and evolution of mental health policies in the future.

**Conclusion:**

This is the first study to discuss and define the challenge of TRD/DTD in Greece and propose a structured pathway for its integrated management in the context of the Greek NHS, allowing for the country’s geographic disparities, history of burden of mental health and socioeconomic specificities, including stigma surrounding a mental health diagnosis.

## 1 Introduction

Major Depressive Disorder (MDD) is a significant public health challenge that gravely impacts patients, carers, and health care systems. The diverse symptoms combinations that qualify for a diagnosis of MDD give rise to symptom variability among individuals diagnosed with the condition. This could potentially lead to a wide range of pharmacologic and non-pharmacologic treatment options for MDD demonstrating varied, and sometimes, limited efficacy (1).

The challenges in treating MDD are further aggravated by a subgroup of MDD patients who do not respond adequately to treatment, despite multiple courses of different treatments, including anti-depressants. This condition is defined as Treatment Resistant Depression (TRD) – a term that has raised concerns, including semantic, such as who is resistant, the disease or the patient (2), and for which over 155 different definitions have been proposed (3). Interestingly, the definition of TRD within clinical research is also variable – only 17% of intervention studies defined TRD based on at least 2 treatment failures (4), emphasizing the variety of TRD definitions in both clinical practice and research.

A different, more “empathic” (2) concept of Difficult to Treat Depression (DTD) has also been proposed (5), which may be considered “more pragmatic, drawing on the models of care for chronic physical health problems with waxing and waning symptoms such as arthritis, diabetes and hypertension, focused on examining the burden of depression in naturalistic clinical practice” (5).

Due to the confusion around the term, TRD or DTD is not only difficult to treat; it is also difficult to identify. Who are the patients that may qualify as “treatment resistant” or “difficult to treat”? What are their characteristics? Can we profile them to assist with their swift identification? And if we can agree on their profile, how do we then manage their condition? Currently, there is no established Standard of Care (SoC) for TRD, and there is wide variation in clinical approaches to overall disease management (6, 7).

Most of these, stem from the lack of consensus on a series of elements included in the definition of TRD or DTD, such as:

- What can qualify as anti-depressant “failure”?
- How can treatment “response” be realistically assessed in clinical practice?
- What are the elements that make up such a response and failure, including target dosing, duration of treatment, tolerability, and adherence?
- How many treatment failures are required to establish treatment resistance/difficulty in treating? Do they refer to the same or different MDDs? Should treatments be from different anti-depressant classes? Should we include psychotherapy or neurostimulatory treatments?
- What are the goals of treatment in such patients? Do they differ from general depression treatment goals?
- How are principles of management affected by the nature of TRD/DTD?
- Is there a need for integrated service pathways, so that patients do not fall between the cracks of specialist outpatient and inpatient care and broader primary care?
- How would such pathways be organized and implemented to address the unmet need in patients with TRD/DTD and support care providers and patients with successfully managing the condition?

This consensus document brings together clinical and health policy expert opinions on (a) the characteristics of TRD/DTD patients, to help with patient profiling, i.e., with the swift and accurate identification of patients that may be eligible to follow the TRD/DTD treatment pathway, and (b) the definition of an optimal pathway for the management of TRD/DTD in Greece, allowing for the specificities of the Greek health care system.

## 2 Methods

Individual interviews with the experts were conducted in November 2022, based on a structured interview guide that included close-ended questions and free-text boxes after each question (please see Supplement) to invite additional comments and perspectives. The clinical expert group consisted of six psychiatrists with 15 to 39 years of experience, primarily working in hospital settings (general and psychiatric hospitals) across Greece, including Athens, Thessaloniki, Ioannina, and Larissa (details in **Table 1**). All experts hold academic positions at leading Greek universities, reflecting a highly experienced and geographically diverse group with expertise in both patient care and research. The experts were a convenient and representative sample of professionals with specific expertise in treating depression, particularly Treatment-Resistant Depression (TRD). They were selected based on their extensive experience, which encompassed both inpatient and outpatient care. No specific inclusion or exclusion criteria were applied, as the goal was to gather insights from a diverse group of leading experts in the field.

**Table 1:** Consensus findings on (a) the characteristics of TRD/DTD patients.

Findings	Consensus*	Comments/Notes
Definition of health state as DTD versus TRD	Y	TRD may be more appropriate for wider communication of the criteria that need to be present to correctly diagnose/characterize such patients and reach a shared decision on a treatment strategy
Characteristic elements of TRD/DTD:		
Failure defined as inadequate/diminished response	Y	
Failure defined as not achieving remission	Y	
Assessment of response/failure (MADRS, CGI)	Y	
Assessment of response/failure (BDI, PHQ-9)	Y	
Optimal treatment dose defined as upper limit of dose range (label)	Y	Conditioned by specific patient characteristics
Optimal treatment duration (4–6 weeks)	Y	Note on possibility of earlier or later assessment, based on personal circumstances
Adherence to treatment (hardest element to validate). Based on discussion with patient and carer as well as the development of a sincere, trustful relationship that addresses any misconceptions and helps strengthen adherence	Y	
2# failed trails before establishing difficulty to treat/treatment resistance, including augmentation with a second antidepressant or other psychiatric medication. Psychotherapy is considered a pre-requisite, but it is excluded from augmentation calculations	Y	
All qualifying trials should occur within the same episode	Y	
Primary goal of treatment in TRD/DTD is the optimization of psychosocial functioning and the return to meaningful life	Y	
Other goals include reduction in risk and impact of relapse and optimal symptom control	Y	
Overall guiding principle of treatment in TRD/DTD is to enhance engagement and retention in care and treatment	Y	
This includes sustained shared decision making with patient and carers, and enhancing the use of self-management and coping strategies, particularly during crises	Y	
Secondary guiding principle of treatment in TRD/DTD is the continuous re-assessment of treatment direction	Y	
Continuous re-assessment of treatment direction includes monitoring and measuring for severity of symptoms and their impact on psychosocial functioning	Y	
It also includes regular and consistent review of initial diagnosis and should account for precipitating, predisposing and perpetuating factors that may impact on actual diagnosis and patient engagement in treatment	Y	

* based on majority of responses, #=number.

BDI, Beck’s Depression Inventory; CGI, Clinical global impression scale; DTD, Difficult-to-Treat-Depression; MADRS, Montgomery–Åsberg Depression Rating Scale; PHQ-9, Patient Health Questionnaire-9 item; TRD, Treatment Resistant Depression; Y, Yes.

The interview guide was developed specifically to produce a consensus on the questions outlined in the introduction. It was prepared by the Health Policy Institute in English, and the interviews were conducted in Greek using paper and pen. Prior to the interview, the interview guide was shared with all experts, who were asked to fill in the guide and return to the Health Policy Institute. During the scheduled interview, discussion was facilitated by a health policy professor and two researchers from the Health Policy Institute, all of whom had extensive experience in conducting such interviews. Responses already submitted to the interview guide were discussed in depth, and responses to unanswered questions were collated during the interview. All responses were validated with each interviewee at the end of the interview. Responses were then translated into English during data entry. Tables with all interviewee responses to each question were prepared to confirm consensus with each statement/question. Where additional free text was provided by an interviewee, it was tabulated next to the question as additional input to be considered during the preparation of the consensus manuscript draft. Positive responses (Yes, Y) to the statement/question were counted and, in the cases of majority (all cases), a Y added to two separate Consensus Tables, one on the characteristics of TRD/DTD patients (**Table 1** below) and one on the definition of an optimal pathway for the management of TRD/DTD in Greece (**Table 2** below). A draft manuscript was written and circulated among the authors, with revisions made iteratively based on feedback. This final document reflects broad agreement on principles to which all authors could subscribe.

**Table 2:** Consensus findings on (b) the definition of an optimal pathway for the management of TRD/DTD in Greece.

Findings	Consensus*	Comments/Notes
Making care services for patients with TRD/DTD easily accessible, particularly for areas of Greece with no geographic proximity or easy access to mental health hospitals, or mental health clinics within general hospitals, and/or tertiary hospitals, is a priority for the health care system in Greece	Y	Developing integrated/comprehensive networks of care with local reach and centralized guidance
Such an integrated network would include at least:		
A (referring) psychiatrist from the local general health services (NHS/contracted EOPYY).	Y	
A TRD/DTD Clinic in tertiary hospitals	Y	
Mobile mental health units	Y	
Standard of Care for care provision	Y	
Digital tool for integrating & monitoring process	Y	
The benefits of such a network would include:		
Patient Centered care	Y	
Accessibility	Y	
Affordability	Y	
Evidence-based decision making	Y	

DTD, Difficult-to-Treat-Depression; EOPYY, National Organization For Health Care Services; NHS, National Health System; TRD, Treatment Resistant Depression; Y, Yes.

## 3 Results – consensus

Most of the consensus group participants preferred the use of the DTD term over TRD. TRD was considered a term that lacked empathy and did not adequately reflect clinical reality, whereas DTD was perceived as a more open concept that could accommodate a more pragmatic representation of the difficulties involved in managing such cases in clinical practice. Nonetheless, consensus group participants noted that the term TRD may be easier to use as reference amongst wider groups of clinicians, as it draws on the much-referenced concept of resistance.

Almost all consensus group participants agreed on the need to set bold treatment goals, as well as ensure that these are not met, before defining a case as “difficult to treat”. For instance, all participants agreed that difficulty to treat or, alternatively, treatment resistance should be established only when the following criteria are cumulatively present (8, 9):

- Failure
- To respond
- To ≥2 anti-depressant therapies
- Given at adequate doses
- For an adequate duration
- During one major depressive episode (moderate to severe)

The issue of failure to respond was extensively discussed during the interviews. Most authors agreed that a response, such as a 50% reduction in symptom rating scales like the Hamilton Depression Scale (HAMD) or the Montgomery-Åsberg Depression Rating Scale (MADRS), may represent a desirable clinical outcome. However, it cannot be considered a definitive treatment goal. Instead, it is more akin to a “desperation” goal. They all agreed that the definitive element of failure or lack thereof of a treatment is the ability of the patient to be functional, i.e., to return to work or school and resume normal activities. On the other hand, three out of five authors agreed with the statement that failure to achieve remission, defined according to DSM-V criteria as a failure to achieve ≥2 months with no symptoms or only one or two symptoms to no more than a mild degree and confirmed by a clinician-rated tool such as the CGI, which is easier to fill in and relies on clinical judgement, HAM-D, or MADRS, which is the tool of choice by all authors, should be sufficient to substantiate a treatment failure. All authors stressed the important of “correct” diagnosis, before the assessment of any failure to respond to treatment, including the exclusion of the presence of any comorbidities, e.g., more specifically anxiety and bipolar disorder.

This level of response or remission needs to be measured. All consensus group participants agreed that the use of clinical tools, such as the easy to administer CGI or the more elaborate and potentially more time-consuming but equally more valid MARDS, are critical in assessing response. Nonetheless, they all noted that psychiatrists in everyday clinical practice may lack the time or the experience to use these scales and tools, particularly outside the context of clinical trials. It is essential that questionnaires, tools, and scales used to assess response to treatment are easy to administer and as short as possible, particularly if to be applied outside of experienced Centers of Excellence or University Clinics. They underlined the importance of agreeing on a simple, easy to administer tool to systematically record and assess response to treatment, including patient self-assessment tools such as the BDI and the PHQ-9, which is easy to use, even in the private psychiatrist setting, and can provide insights into the status of the patient, thus informing the therapeutic session. The authors strongly support the need to maintain all these tools in a digital format that can be completed online and saved onto the patient’s clinical record, to use during psychotherapy as well as to monitor progress through time.

In each clinical evaluation of response or failure, all authors agreed that the following elements should be assessed and accounted for:

- *Treatment dose*. According to all consensus participants, response or failure should be addressed only after the optimal treatment dose is achieved as part of “adequate” treatment. This optimal dose is, according to 4 out of 5 clinical experts participating in this consensus panel, the upper limit of dose according to label of each treatment, unless there are specific side effects or genetic challenges that prevent a patient from receiving upper limit of dose as per label.
- *Treatment duration*. This was the element of treatment to be assessed that appears to invite the greatest debate, elsewhere and in Greece. Expert opinions in this consensus group ranged from <4 weeks to between 4 and 6 weeks, in cases where the concurrent life circumstances of the patient would make an expectation of response in less than 6 weeks highly unlike, e.g., loss of family member, loss of employment, divorce etc. A reference to a shorter period of 2–4 weeks was also substantiated by recent literature that underlined the lack of the luxury of time in specific cases amongst treatment resistant/difficult to treat patients.
- *Adherence to treatment*. This is the element of treatment that all experts agreed is the hardest to validate. From electronic pill counts to digital applications monitoring adherence to treatment, it is accepted that comprehensive and sincere reporting of non-adherence by the patient and/or carer is probably unrealistic in actual clinical practice in Greece and can only be based on the depth of trust to be established in the relationship between patient and therapist. Psychoeducation and improved access to therapeutic drug monitoring may have a positive impact on adherence. The most critical element of achieving optimal adherence is, all clinical experts agreed, to ensure trust is established between the patient and the therapist, so that the patient can freely discuss thoughts on adverse events’ burden, fear of taking the medication as instructed, as well as any other factors that may negatively impact on adherence. A participant in the consensus group stressed the importance of evaluating the presence of any comorbidities up front, e.g., stress disorders, as they may aggravate the challenges related to adherence.

All participants agreed on a minimum of 2 previous trials of antidepressants before establishing treatment resistance or difficulty to treat in depression. Nonetheless, it was noted that in certain cases, resistance may be established after the trial of a single antidepressant, provided there has been augmentation with a second antidepressant, which has been added to the first one for an adequate time. One of the clinical experts noted that trials should include at least one SSRI and one SNRI and one add-on therapy. All consensus group participants agreed that treatment is based on addition of therapeutic options until response is achieved, rather than switching between options, once a failure is recorded. All participants considered psychotherapy and Cognitive Behavioral Therapy (CBT) as an integral part of treatment from the start and not a part of “augmentation” as used for the definition of trials required to fail to establish the resistance definition – one even noted that augmentation may refer to simply an increase in frequency of psychotherapy sessions.

All participants recognized the clinical benefits of Electro Convulsive Therapy (ECT) in the treatment of resistant depression, assuming there is capacity in general hospitals to provide such services. This capacity would include the presence of an anesthesiologist, as well as the availability of an Intensive Care Unit (ICU) bed to manage any treatment-related challenges. Yet, a disparity between Athens and the rest of Greece was noted, as outside Athens, no public hospital offers ECT to patients suffering from a psychiatric disorder. Repetitive Transcranial Magnetic Stimulation (rTMS) is another brain stimulation method that has demonstrated efficacy in TRD/DTD, but it is not widely available in many parts of the country, mainly due to its high cost.

In any case, all participants agreed that resistance or response should be established during the same MDD episode. However, one participant noted that the length of this episode should be defined to assist with the definition and management of the condition.

Further and as regards TRD/DTD management, all clinical experts agreed that the goal of treatment in TRD/DTD is the optimization of psychosocial functioning and the return to meaningful life. Secondary goals were reduction in risk and impact of relapse and optimal symptom control. They also, all, agreed that the overall guiding principle of treatment in TRD/DTD is to enhance engagement and retention in care and treatment, followed by the continuous re-assessment of treatment direction.

Enhancing engagement and retention in care for all clinical experts was construed as primarily including sustained shared decision making with patient and carers. They all stressed the importance of patient empowerment, which in the clinical setting actively recognizes and promotes the patient role in the individual long-term management plan and the need to recognize patient choices and discuss and incorporate patient preferences in such a plan, if to achieve treatment goals. All experts equally stressed the importance of self-management strategies, particularly during crises. Encouraging good habits, enhancing ability to cope with residual symptoms and behavioral activation, all contribute to meeting treatment goals and are critical elements of strengthening patient resilience in successfully managing his or her condition.

Continuous re-assessment of treatment direction was agreed by all participants to include monitoring and measuring for severity of symptoms and their impact on psychosocial functioning to evaluate whether such symptoms may point to a varied or blurred diagnosis. This would evolve in tandem with regularly and consistently revisiting the initial diagnosis, allowing for any screening of comorbidities that impact on treatment outlook and patient engagement in treatment to be accounted for and actively managed. Any precipitating, predisposing and perpetuating factors should also be accounted for, and their impact measured both on actual diagnosis and on patient attitude towards treatment, to ensure optimization of treatment approach and expected (and realized) treatment outcomes.

Finally, as regards the optimal pathway to manage TRD/DTD within the Greek National Health System, allowing for its specificities, and particularly the geographic disparities between, and within, regions, all participants recognized that treatment provision in TRD/DTD includes therapeutic options that can only be delivered within the hospital setting, or under supervised care. Making care services for patients with TRD/DTD easily accessible, particularly for areas of Greece with no geographic proximity or easy access to mental health hospitals, or mental health clinics within general hospitals, and/or tertiary hospitals, is -to all clinical experts participating in the consensus panel- a priority for the health care system in Greece. Networking the treating physician and the patient as a “team” with a Center of Excellence on TRD/DTD, instead of asking the patient to choose between continuing to receive treatment at the point of care that he/she currently is or move to a different setting, most probably in a locality that is remote from place of residence, only to be able to access optimal care, would ensure continuity in disease management at the point of care that is most convenient for the patient. It would also sustain inclusion and ownership of case by the treating physician, even as treatment approaches and options evolve and intensify to offer the patient an optimal pathway relative to his/her needs.

Such a network would need to include the following key collaborating roles and facilities:

- A (referring) psychiatrist from the local general health services (NHS/contracted EOPYY). The referring psychiatrists remain involved in the care pathway post referral. They are also responsible for monitoring treatment progress and patient response during follow up between treatments for TRD/DTD. They, thus, remain engaged with the patient and his/her treatment pathway and become a part of an integrated solution that does not remove the patient from their care, but on the contrary, reinforces the therapeutic relationship between the physician and the patient, as additional and potentially more appropriate treatment options are becoming available to patients.
- A TRD/DTD Clinic in tertiary hospitals. These clinics act as centers of excellence and accept referrals from psychiatrists as well as train and supervise the operation of the mobile mental health units (described in detail below).
- Mobile mental health units allocated to regions across the country, linked to and supervised by the respective tertiary hospital TRD/DTD Clinic. Mobile health units provide care to TRD/DTD patients, according to an agreed treatment schedule, are operated by an overseeing psychiatrist and a specialized nurse (and a driver) and are equipped with all required infrastructure, including technological, to support care delivery according to clinical guidelines and the various treatments’ Risk Management Plan.
- The latter may be complemented with the Centers for Mental Health that are being deployed or upgraded across the country as part of the most recent mental health initiatives to integrate mental health services with primary care and the general health services. These Centers for Mental Health will be already adequately staffed and depending on geographic position in catchment area can cater to the needs of the network, with additional infrastructure as appropriate.

Such a network would operate based on a Standard of Care (SoC) – i.e., an agreed process to describe the minimum common elements of the care process, to integrate the various parts of the network and to define how, when and using which tools they should interact. The SoC describes a specific process and process steps and would also include a set of Key Performance Indicators (KPIs) against which the process for care provision may be audited. Standardization of care constitutes a critical prerequisite for assuring the quality-of-care provision across different health care facilities, in different geographies, to the benefit of patient experience and outcomes and system efficiency. When coupled with digitalization and digital integration of patient pathways, it allows for rapid adjustments to changing needs and circumstances, including through real time monitoring and auditing of its measurable outputs.

Nonetheless, all participants agreed that the implementation of SoC would be contingent upon the effective introduction of a digital tool that would monitor care provision inside the network. The tool or platform would need to be accessible by the different users according to their role and would be used to optimize data sharing, appointment scheduling (particularly for mobile units) and epidemiological monitoring, including e.g., prevalence of TRD/DTD, concentration across geographies in the country, level of coverage, and unmet needs etc.

The benefits of establishing such an integrated network of care would include:

- Patient-centered care. The network works around the needs and the specific circumstances of the patient to truly customize the care experience, bringing the care to patients, and enhancing adherence and engagement. It also facilitates retention in care, as it removes access barriers related to geography and supports seamless integration with and minimal disruption of patient’s and family’s life.
- Accessibility. Including geographic proximity, of the complete range of treatment solutions across the country. Making the range of appropriate services adequately accessible to patients in their place of residence or with minimal travel is key not only in reducing travel burden and logistics for the patient but also minimizing the financial toxicity of care provision for the household – and can thus contribute to enhanced retention in care.
- Affordability. Relevant to the previous points, offering specialized care both in tertiary clinics and in mobile units/Centers for Mental Health, as appropriate, ensuring no additional burden (travel, accommodation, time away from work or home) for the patient and carer contributes to greater affordability of those services and thus increases the probability of retention in care and adherence to treatment.
- Evidence-basis for future interventions. This includes improved mapping of disease epidemiology, concentration across geographies/other factors, level of coverage of services, including e.g., requirements in resources, infrastructure, etc. (gap identification).

## 4 Gap analysis for the Greek health care setting

Introducing such a network would require some structural changes in the NHS, particularly to address the legacy gaps in integration of mental health services with primary and general health services.

Firstly, it would require the establishment or re-allocation of existing mobile mental health units that should meet at least the following standards to be able to provide targeted treatment and care provision for TRD/DTD:

- An appropriate vehicle. It should be possible to dim the lights inside the vehicle (e.g., draw curtains) and isolate any noise/distractions. It may also be helpful to include infrastructure for cognitive stimuli (e.g., ambient lighting and/or imagery) (10). The vehicle should be discrete and care provision should be shielded from external view. It may also need to include a toilet.
- A reclining seat. A comfortable seat that may recline to 45 degrees, where patients may be examined and/or receive treatment and care.
- Basic medical equipment such as blood pressure, oxygen saturation, temperature, height, and weight measuring devices.
- Specialized medical equipment: electrocardiogram device, automated external defibrillator, portable oxygen generators. This is especially relevant when treating patients with clinically significant or unstable cardiovascular or respiratory risk factors. Psychiatrist or specialized nurse on board the mobile unit should be trained in cardiopulmonary resuscitation.
- Medication for managing possible side-effects and for providing a first aid response.
- PC/laptop with access to the internet, to be able to access and use the digital tool for monitoring care provision, including to schedule appointments etc.

It is stipulated that a mobile unit will be made available to each reference tertiary hospital with a university psychiatry clinic (Center of Excellence) that will organize and supervise its function across the relevant catchment area. As mentioned above, the mobile unit will need to be manned with a psychiatrist, nurse and driver, and these roles may rotate among more individuals to cover the relevant shifts. Number of shifts and distribution of appointments in the catchment area would be defined by supervising Center of Excellence, which will be also responsible for providing training to participating staff. To perform these tasks the supervising Center of Excellence should be allocated with a Project Manager, who will be responsible for ensuring: a) smooth operation of mobile unit as per plan, b) coordination between Center of Excellence and unit, c) availability of consumables and d) effective management and resolution of challenges arising from the operation of the mobile unit.

Relevant to the mobile mental health unit, the integration of Centers for Mental Health in the network may require appropriate infrastructure being made available, including seats/beds, medical equipment, medication, and technology infrastructure listed above for mobile units. Centers for Mental Health would be treated essentially as “outposts” of the tertiary Center of Excellence, for care provision in TRD/DTD, and could be further integrated with any available mobile units in the same catchment area, to avoid overlaps and optimize efficiency.

Equally, to ensure availability of ECT, where and if clinically appropriate, particularly in Athens, consensus participants from Athens stressed the need to mobilize hospital resources to ensure the availability of such a service. This would include the allocation of an anesthesiologist to the ECT service for the days that such services will be provided, as well as the reservation of other hospital related facilities (e.g., ICU beds) to manage any potential adverse events or eventualities. A schedule for the operation of the service would have to be drawn based on need and the hospital management should have the flexibility to re-allocate resources internally to cater to the needs of the service.

Even if all infrastructure and human resources are put in place for the operation of such a network of integrated care, it would be to no avail, should there be no referrals of eligible patients from treating psychiatrists. It is imperative that referral pathways are fully detailed and cover the totality of potential entry points of the patient in the network, including, for instance, primary care centers or personal doctors that may be treating or to which a patient turns at the first instance, thus ensuring full integration of the service in “normalized” care provision. Apart from increasing the acceptability of the service and removing any elements of stigma, such an integration would be extremely beneficial in supporting the earlier identification of potential cases. To this end, it is imperative that this initiative is complemented by education of General Practitioners (GPs), psychiatrists in private practice and in district general hospitals, psychologists, social workers etc. on the importance of prompt referrals for the effective and efficient management of TRD/DTD. This referring network could further be trained and empowered to perform basic screening and diagnostic services, including imaging investigations and neuropsychological assessment, so that they a) become involved and engaged in the overall treatment management process from very early on and b) they feel empowered to further integrate with the network and advocate for its use, as necessary.

## 5 Discussion

This study is the first effort of clinical and health policy experts to openly discuss and attempt to define the challenges of Treatment-Resistant Depression TRD/DTD in Greece. Our findings align with international guidelines and agencies on TRD/DTD, particularly the definitions provided by the US Food and Drug Administration (FDA) (11) and the European Medicines Agency (EMA) (12), which consider TRD as the failure to respond to two or more antidepressant regimens despite adequate dose, duration, and adherence to treatment. However, it is important to note that the absence of a universally accepted and validated definition of TRD remains a significant limitation, both in Greece and globally (13). This lack of consensus impacts not only research, but also treatment development, and clinical and policy decision-making. In this study, all authors stressed the importance of a “correct” diagnosis before assessing any failure to respond to treatment. This includes the exclusion of bipolar depression and the assessment of comorbidities, such as anxiety, dementia, substance use disorders, or somatic disorders.” Overall, our consensus is in line with a previous one published by McAllister-Williams (2), particularly with regards to goals and guiding principles. Nonetheless, by contextualizing our results within this broader international framework, we highlight the shared challenges in defining and managing TRD/DTD, while also underscoring the need for standardized criteria to guide research, clinical practice, and policy initiatives worldwide.

Furthermore, our recommendation for integrating mobile mental health units, Mental Health Centers, and tertiary Centers of Excellence offers a promising framework for improving access to mental health care. As Greece has been plagued by a persistent economic crisis, which has resulted in a severe burden in terms of mental health, it is critical that these challenges are urgently and adequately addressed. This is even more critical in the aftermath of the COVID-19 pandemic and the ongoing energy and economic crisis, which are expected to exacerbate mental health issues globally and in Greece. Mental health services in Greece have traditionally been underfunded and further strained by the economic crisis, which delayed psychiatric reform due to austerity measures and prioritized cost-cutting (14). During the crisis, community mental health services faced increased pressure due to rising patient needs, particularly for psychotherapeutic interventions and psychological support. Higher unemployment rates also affected the influx of new patients and the therapeutic management of existing ones. Reinforcement of the community mental health service network has been identified as a key strategy to mitigate the crisis’s impact on population mental health (15).

The situation is graver in underserved areas of the country, which are situated at a distance from large urban centers. Health policy experts and analysts have identified deficiencies in the access and provision of the mental health services in remote and inaccessible areas, in Greece (16, 17). For those areas, integration of care services at the point of need, including through provision of services via mobile units, has been shown an effective solution to meet patient needs, particularly in mental health, where Lykomitrou et al. (18) estimated that deployment of Mobile Mental Health Units in the Cyclades Islands averted 17.98 Disability-Adjusted Life Years (DALYs) in 2015. In addition, the MMHU I-T (Mobile Mental Health Unit Ioannina – Thesprotia) has become an integral part of the local primary care system and is well known to the population of the catchment area. By the end of 2016, 60% of patients were self-referred or family-referred, compared to 24% in the first 2 years. In 2017, the number of active patients was 293 (mean age 63 years, 49.5% are older adults), and the mean caseload for each member of the team is 36.6. A significant proportion of patients (28%) received care with regular domiciliary visits, and the provision of home-based care was correlated with the age of the patients. Within the first 2 years of operation of the MMHU I-T hospitalizations of treatment, engaged patients were reduced significantly by 30.4%, whereas the treatment engagement rates of patients with psychotic disorders were 67.2% in 5 years. The MMHU I-T and other similar units in Greece are a successful paradigm of a low-cost service which promotes mental health in rural, remote, and deprived areas (16).

In line with these encouraging findings, and to further address geographic disparities, the proposed framework emphasizes the expansion of mobile units and the establishment of localized Mental Health Centers. Additionally, to combat stigma, the framework incorporates community-based education and awareness programs delivered through these centers and mobile units. Collaboration with local organizations and stakeholders is also encouraged to normalize mental health care and reduce cultural barriers. By combining accessible service delivery with targeted anti-stigma initiatives, the framework aims to create a more inclusive and equitable mental health care system.

However, practical barriers such as workforce shortages, financial constraints, and policy limitations must be acknowledged and addressed. For instance, the availability of trained mental health professionals, particularly in rural or underserved areas, remains a significant challenge. Financial resources are often limited, and sustainable funding mechanisms will be essential to support the infrastructure and operational costs of these integrated services. Additionally, policy reforms may be needed to facilitate collaboration across different levels of care and ensure the effective implementation of these models. By proactively addressing these barriers, stakeholders can work toward a more equitable and efficient mental health care system.

Overall, our study highlights the urgent need for policy reforms that prioritize the integration and expansion of mental health services, particularly for TRD/DTD. This includes increasing funding for community-based care, strengthening mobile mental health units, and addressing workforce shortages. Additionally, the study underscores the importance of addressing stigma and geographic disparities through targeted education and outreach programs. By implementing these recommendations, Greece can build a more resilient and equitable mental health care system capable of addressing current and future challenges.

## 6 Limitations

This consensus document is not based on a formal process, such as the Delphi technique. The reasons for this are that all authors shared from the outline of the project a strong and shared consensus on a very broad range of questions to be addressed during the interviews, such that would render the Delphi process not warranted. During the iterative process of producing the manuscript, there was very little disagreement between authors, which is noted in the respective sections. All contributors endorsed the outcomes of this iterative process by signing off the manuscript and agreeing to be identified as authors.

## Data Availability

The original contributions presented in the study are included in the article/**Supplementary Material**. Further inquiries can be directed to the corresponding author.
