# Supplementary material for: Treatment-resistant or difficult-to-treat depression: a consensus on the pharmacotherapy challenges and considerations for the health care system in Greece
Source: Front Psychiatry. 2025 Apr 28;16:1561821. doi: 10.3389/fpsyt.2025.1561821 (PMC12066590; doi:10.3389/fpsyt.2025.1561821)
Supplement: Supplementary file 1 [file DataSheet1.pdf]

Agreeing on an optimal disease management pathway for TRD in Greece

Interview Guide

1. Introduction – scope of the interview

Major Depressive Disorder (MDD) is a significant public health challenge that gravely impacts patients, carers, and health care systems. The diverse symptoms combinations that qualify for a diagnosis of MDD give rise to symptom variability among individuals diagnosed with the condition. This could potentially lead to a wide range of pharmacologic and non-pharmacologic treatment options for MDD demonstrating varied, and sometimes, limited efficacy (Han et al. 2020).

The challenges in treating MDD are further aggravated by a subgroup of MDD patients who do not respond adequately to treatment, despite multiple courses of different treatments, including anti-depressants. This condition is defined as Treatment Resistant Depression (TRD) – a term that has raised concerns, including semantic (who is resistant? the disease or the patient? McAllister-Williams et al. 2020) and for which over 155 different definitions have been proposed (Brown et al. 2019). Interestingly, the definition of TRD within clinical research is also variable – only 17% of intervention studies defined TRD based on at least 2 treatment failures (Gaynes et al. 2020), emphasizing the variety of TRD definitions in both clinical practice and research.

A different, more "empathic" (McAllister-Williams et al. 2020) concept of Difficult to Treat Depression (DTD) has also been proposed (Costa et al. 2022), which may be considered "more pragmatic, drawing on the models of care for chronic physical health problems with waxing and waning symptoms such as arthritis, diabetes and hypertension, focused on examining the burden of depression in naturalistic clinical practice" (Costa et al. 2022).

Irrespective of the term to be used, but, in essence, due to the confusion around the term, TRD or DTD is not only difficult to treat; it is also difficult to identify. Who are the patients that may qualify as "treatment resistant" or "difficult to treat"? What are their characteristics? Can we profile them to assist with their swift identification? And if we can agree on their profile, how do we then manage their condition? Currently, there is no established Standard of Care (SoC) for TRD, and there is wide variation in clinical approaches to overall disease management (Perugi et al. 2021).

Most of these, stem from the lack of consensus on a series of elements included in the definition of TRD or DTD, such as:

- What can qualify as anti-depressant "failure"?
- How can treatment "response" be realistically assessed in clinical practice?
- What are the elements that make up such a response and failure, including target dosing, duration of treatment, tolerability, and adherence?
- How many treatment failures are required to establish treatment resistance/ difficulty in treating? Do they refer to the same or different MDDs? Should treatments be from different anti-depressant classes? Should we include psychotherapy or neurostimulatory treatments?
- What are the goals of treatment in such patients? Do they differ from general depression treatment goals?
- How are principles of management affected by the nature of TRD/DTD?

- Is there a need for integrated service pathways, so that patients do not fall between the cracks of specialist outpatient and inpatient care and broader primary care?
- How would such pathways be organized and implemented to address the unmet need in patients with TRD/DTD and support care providers and patients with successfully managing the condition?

This interview aims to elicit clinical expert opinions on:

(a) the characteristics of TRD patients, to help with patient profiling, i.e., with the swift and accurate identification of patients that may be eligible to follow the TRD treatment pathway and

(b) the definition of an optimal pathway for the management of TRD in Greece, allowing for the specificities of the Greek health care system.

You are kindly invited to answer the following questions and provide any additional input in the free text boxes. Your answers will be collated, analyzed, and synthesized into a consensus statement to be returned to you for your comments, in preparation for an Expert Consensus Panel to discuss findings and agree on priorities for action.

2. Characteristics of TRD /DTD

You are kindly invited to answer the questions that follow. Please add any further thoughts and comments in the free text box below each question.

2.1 Would you agree that the following elements should be included in the definition of TRD and explicitly explained, as below*?

Element	Include Yes/No	If no, what should we include instead?
Failure		
To respond		
To ≥2 anti-depressant therapies		
Given at adequate doses		
For an adequate duration		
During one major depressive episode (moderate to severe)		

*EMA's definition of TRD, 2013, 2019

Additional thoughts and comments

2.2 What in your opinion are the clinical criteria for antidepressant "failure"? When should we conclude that an anti-depressant treatment has "failed"?

Element	Yes	No
No response at all		
Inadequate/diminished response, e.g., <50% improvement in the severity of depressive symptoms or an inability to return to work/study, to be confirmed by a clinician-rated tool such as the CGI, HAM-D or MADRS		
Failure to achieve remission, defined according to DSM-V criteria as a failure to achieve ≥2 months with no symptoms or only one or two symptoms to no more than a mild degree and confirmed by a clinician-rated tool such as the CGI, HAM-D or MADRS		
Other. Please detail/explain below		

Additional thoughts and comments

2.3 How should "response" be assessed in clinical practice?

**Element**	**Yes**	**No**
Clinical judgement		
Use of clinical tools*. Please indicate if yes/no and preference for which of the below. Please add more tools, if appropriate<br>• BDI (Beck Depression Inventory)<br>• CGIs<br>• DSM<br>• HAMD-17<br>• HDRS<br>• MADRS<br>• Mental State Examination (MSE)		
Validated patient self-rated scales*<br>• Zung Self-Rating Depression Scale (SDS)<br>• Patient Health Questionnaire 9 (PHQ-9)<br>• QIDS-16<br>• EuroQol visual analogues scales (EQ-VAS)<br>• Mental State Examination (MSE)		

* As discussed in Han et al. 2020, Kasper et al. 2020

Additional thoughts and comments

2.4 What are the elements that make up response or failure and should be accounted for in the clinical evaluation?

**Element**	**Yes**	**No**
Treatment dose		
Treatment duration		
Adherence to treatment		
Other. Please detail/explain below.		

Additional thoughts and comments

2.5 Specifically with regards to treatment dose, should we assess failure/response only after achieving optimal dose as part of adequate treatment?

*Please answer this question ONLY if you have answered **Yes** to Treatment dose in question 2.4 above*

**Element**	**Yes**	**No**
Achieve optimal dose as part of adequate treatment		
What is an "optimal" dose? Please select one of the below		
• Upper limit of dose according to label		
• Within the dose range according to label		
• Lower limit of dose according to label		
• Maximum tolerated dose above the range indicated by label		
• Other. Please detail/explain below.		

Additional thoughts and comments

2.6 Specifically with regards to treatment duration, should we assess failure/response only after which target treatment duration?

*Please answer this question ONLY if you have answered **Yes** to Treatment duration in question 2.4 above*

**Element**	**Yes**	**No**
Treatment duration before defining condition as treatment failure		
<4weeks		
4 to <6 weeks		
6 to <8 weeks		
8 to <10 weeks		
≥12 weeks		
Other. Please detail/explain below.		

Additional thoughts and comments

2.7 Specifically with regards to adherence to treatment, should we assess failure/response only after we have confirmed optimal adherence to treatment?

*Please answer this question ONLY if you have answered **Yes** to Adherence to treatment in question 2.4 above*

**Element***	**Yes**	**No**
Ensuring treatment adherence before assessing antidepressant failure		
Computerized prescription records (e-prescription)		
Pill counts / electronic pill containers		
Patient self-report and/or self-reported assessments (TAPQ, BMQ, and MMAS)		
Information from relatives/carers		
Other. Please detail/explain below.		

*Informed by Solmi et al. 2021

BMQ = Brief Medication Questionnaire; MMAS = Morisky Medication Adherence Scale, TAPQ = Treatment Adherence Perception Questionnaire

Additional thoughts and comments

2.8 How many antidepressant treatment failures would you say are required before establishing resistance to treatment/difficulty in treating?

**Element**	**Yes**	**No**
Minimum number of treatment failures to establish resistance/difficulty		
1 treatment failure		
2 treatment failures		
3 treatment failures		
4 treatment failures		
>4 treatment failures		
Other. Please detail/explain below.		

Additional thoughts and comments

2.9 Which drug trial qualifies to be included in your definition of "treatment failures" in question 2.8 above?

**Element***	**Yes**	**No**
If first trial, monotherapy with an antidepressant		

If second trial, monotherapy with an antidepressant from the same class		
If second trial, monotherapy with an antidepressant from another class		
Treatment with a single antidepressant + Augmentation with lithium and /or anti-psychotics and/or mood stabilizers		
Treatment with a single antidepressant + ECT		
Treatment with a single antidepressant + psychotherapy		
Combination therapy (at least two antidepressants combined)		
Combination of antidepressants + Augmentation with lithium and /or anti-psychotics and/or mood stabilizers		
Combination of antidepressants +ECT		
Combination of antidepressants +psychotherapy		
Antipsychotic treatment		
Other. Please detail/explain below.		

*Informed by Gillain et al. 2022

Additional thoughts and comments

2.10 Should the qualifying treatment failures occur within the same MDD episode?

**Element**	**Yes**	**No**
Qualifying treatment failures occur within the same MDD episode		

Additional thoughts and comments

3. TRD/DTD management

You are kindly invited to answer the questions that follow. Please add any further thoughts and comments in the free text box below each question.

3.1 What are the goals of treatment in TRD/DTD?

**Element***	**Yes**	**No**
Optimal symptom control		
Reduction in risk and impact of relapse		
Optimization of psychosocial functioning / Return to meaningful life		
Other. Please detail/explain below		

*Informed by McAllister-Williams et al. 2020

Additional thoughts and comments

3.2 What are the guiding principles of treatment in TRD/DTD?

**Element***	**Yes**	**No**
Enhance engagement and retention in care and treatment		
Continuous re-assessment of treatment direction		
Other. Please detail/explain below		

*Informed by McAllister-Williams et al. 2020

Additional thoughts and comments

3.3 If you have answered Yes in "Enhance engagement and retention in care and treatment" in Question 3.2 above, please indicate actions that would qualify to implement this principle

**Element***	**Yes**	**No**
Enhance engagement and retention in care and treatment		
Sustain shared decision making with patient and carers. Emphasize patient role in individual (long term) management plan – Discuss and incorporate preferences		

Support self-management strategies. Encourage good habits, enhance ability to cope with residual symptoms and behavioral activation		
Other. Please detail/explain below		

*Informed by McAllister-Williams et al. 2020

Additional thoughts and comments

3.4 If you have answered Yes in "Continuous re-assessment of treatment direction" in Question 3.2 above, please indicate actions that would qualify to implement this principle

**Element***	**Yes**	**No**
Continuous re-assessment of treatment direction		
Monitor and measure severity of symptoms and impact on psychosocial functioning		
Reconsider diagnosis and screen for comorbidities		
Review predisposing, precipitating and perpetuating factors		
Other. Please detail/explain below		

*Informed by McAllister-Williams et al. 2020

Additional thoughts and comments

3.5 Treatment provision in TRD /DTD includes therapeutic options that can only be delivered within the hospital setting, or under supervised care. Do you agree that making care services for patients with TRD/DTD easily accessible is a priority, particularly for areas of Greece with no geographic proximity/easy access to mental health hospitals, or mental health clinics within general hospitals, and / or tertiary hospitals?

**Yes**	
**No**	

3.6 If you have answered Yes to Question 3.5 above, would you consider the integrated care network described below as appropriate?

Please indicate with an (X) in the column of your preference on the right.

An integrated care network would include the following:

	Yes	No
**A (referring) psychiatrist** from the local general health services (NHS/ contracted EOPYY). The referring psychiatrists remain involved in the care pathway post referral. They are also responsible for monitoring treatment progress and patient response during follow up between treatments for TRD/DTD		
**A TRD/DTD Clinic** in tertiary hospitals. These clinics act as centers of excellence and accept referrals from psychiatrists as well as train and supervise the operation of the mobile mental health units (please see next below)		
**Mobile mental health units** allocated to regions across the country, linked to and supervised by the respective tertiary hospital TRD/DTD Clinic. Mobile health units provide care to TRD/DTD patients, according to the agreed treatment schedule, are operated by an overseeing psychiatrist and a specialized nurse (and a driver) and are equipped with all required infrastructure, including technological, to support care delivery according to clinical guidelines and the various treatments' Risk Management Plan.		
**A digital tool** to monitor care provision inside the network. The tool is accessible by the different users according to their role and is used to optimize data sharing, appointment scheduling (particularly for mobile units) and epidemiological monitoring, including e.g., prevalence of TRD/DTD, concentration across geographies in the country, level of coverage, and unmet needs etc.		
**A Standard of Care** – i.e., an agreed process to describe the minimum common elements of the care process, to integrate the various parts of the network and to define how, when and using which tools they should interact. The Standard of Care would also include a set of Key Performance Indicators (KPIs) against which care provision may be audited.		
Other. Please detail/explain below.		

The benefits of this network would include:

	Yes	No
**Patient-centered care.** The network works around the needs and the specific circumstances of the patient to truly customize the care experience (bringing the care to patients) and enhance adherence and engagement.		
**Accessibility**. Including geographic proximity, of the complete range of treatment solutions across the country.		

**Affordability.** Offering specialized care both in tertiary clinics and in mobile units, as appropriate, ensuring no additional burden (travel, accommodation, time away from work or home) for the patient and carer.		
**Evidence-basis for future interventions**. This includes improved mapping of disease epidemiology, concentration across geographies / other factors, level of coverage of services, including e.g., requirements in resources, infrastructure, etc. (gap identification)		
Other. Please detail/explain below		

Additional thoughts and comments

3.7 If you agree that mobile units may be an appropriate element of the integrated care network, please confirm minimum requirements below

Minimum requirements for the operation of mobile health units include:

	Yes	No
**An appropriate vehicle.** It should be possible to dim the lights inside the vehicle (e.g., draw curtains) and isolate any noise / distractions. It may also be helpful to include infrastructure for cognitive stimuli (e.g., ambient lighting and /or imagery) (Kasper et al. 2020). The vehicle should be discrete and care provision should be shielded from external view. It may also need to include a toilet.		
**A reclining seat.** A comfortable seat that may recline to 45 degrees, where patients may be examined and / or receive treatment and care.		
**Basic medical equipment** such as blood pressure, oxygen saturation, temperature, height, and weight measuring devices		
**Specialized medical equipment:** electrocardiogram device, automated external defibrillator, portable oxygen generators. This is especially relevant when treating patients with clinically significant or unstable cardiovascular or respiratory risk factors. Psychiatrist or specialized nurse on board the mobile unit should be trained in cardiopulmonary resuscitation.		
**Medication** for managing possible side-effects and for providing a first aid response		
**PC/laptop with access to the internet**, to be able to access and use the digital tool for monitoring care provision, including to schedule appointments etc.		
Other. Please detail/explain below.		

3.8 Please list any additional thoughts/concerns that you would like to raise here.

Thank you very much for your input.

References

Brown S, Rittenbach K, Cheung S, McKean G, MacMaster FP, Clement F. Current and Common Definitions of Treatment-Resistant Depression: Findings from a Systematic Review and Qualitative Interviews. Can J Psychiatry. 2019 Jun;64(6):380-387. doi: 10.1177/0706743719828965. Epub 2019 Feb 14. PMID: 30763119; PMCID: PMC6591751.

Costa T, Menzat B, Engelthaler T, Fell B, Franarin T, Roque G, Wei Y, Zhang X, McAllister-Williams RH. The burden associated with, and management of, difficult-to-treat depression in patients under specialist psychiatric care in the United Kingdom. J Psychopharmacol. 2022 May;36(5):545-556. doi: 10.1177/02698811221090628. Epub 2022 May 4. PMID: 35506640; PMCID: PMC9112623.

EMA. 2013. Guideline on clinical investigation of medicinal products in the treatment of depression. [accessed 2020 May 19]. https://www.ema.europa.eu/en/documents/scientific-guideline/guideline-clinical-investigation-medicinal-products-treatment-depression_en.pdf [Google Scholar]

EMA. 2019. Spravato® (esketamine) Summary of Product Characteristics. [accessed 2020 Jan 22]. https://www.ema.europa.eu/en/documents/product-information/spravato-epar-product-information_en.pdf [Google Scholar]

Gaynes BN, Lux L, Gartlehner G, Asher G, Forman-Hoffman V, Green J, Boland E, Weber RP, Randolph C, Bann C, Coker-Schwimmer E, Viswanathan M, Lohr KN. Defining treatment-resistant depression. Depress Anxiety. 2020 Feb;37(2):134-145. doi: 10.1002/da.22968. Epub 2019 Oct 22. PMID: 31638723.

Gillain B, Degraeve G, Dreesen T, De Bruecker G, Buntinx E, Beke D, Kestens C, Valassopoulou E, Verhelst F, Peeters E, Pype S, De Vos C, Strens D, Vandersmissen I. Real-World Treatment Patterns, Outcomes, Resource Utilization and Costs in Treatment-Resistant Major Depressive Disorder: PATTERN, a Retrospective Cohort Study in Belgium. Pharmacoecon Open. 2022 Mar;6(2):293-302. doi: 10.1007/s41669-021-00306-2. Epub 2021 Nov 15. PMID: 34782984; PMCID: PMC8864045.

Han C, Wang G, Chan S, Kato T, Ng CH, Tan W, Zhang L, Feng Y, Liu CY. Definition and Identification of Patients with Treatment-Resistant Depression in Real-World Clinical Practice Settings Across Asia. Neuropsychiatr Dis Treat. 2020 Dec 3;16:2929-2941. doi: 10.2147/NDT.S264799. PMID: 33311981; PMCID: PMC7725069.

Kasper S, Cubała WJ, Fagiolini A, Ramos-Quiroga JA, Souery D, Young AH. Practical recommendations for the management of treatment-resistant depression with esketamine nasal spray therapy: Basic science, evidence-based knowledge and expert guidance. World J Biol Psychiatry. 2021 Jul;22(6):468-482. doi: 10.1080/15622975.2020.1836399. Epub 2020 Nov 12. PMID: 33138665.

McAllister-Williams RH, Arango C, Blier P, Demyttenaere K, Falkai P, Gorwood P, Hopwood M, Javed A, Kasper S, Malhi GS, Soares JC, Vieta E, Young AH, Papadopoulos A, Rush AJ. The identification, assessment and management of difficult-to-treat depression: An international consensus statement. J Affect Disord. 2020 Apr 15;267:264-282. doi: 10.1016/j.jad.2020.02.023. Epub 2020 Feb 7. PMID: 32217227.

Perugi G, Calò P, De Filippis S, Rosso G, Vita A, Adami M, Ascione G, Morrens J, Delmonte D. Clinical Features and Outcomes of 124 Italian Patients With Treatment Resistant Depression: A Real-World, Prospective Study. Front Psychiatry. 2021 Nov 5;12:769693. doi: 10.3389/fpsyt.2021.769693. PMID: 34803777; PMCID: PMC8603563.

Solmi M, Miola A, Croatto G, Pigato G, Favaro A, Fornaro M, Berk M, Smith L, Quevedo J, Maes M, Correll CU, Carvalho AF. How can we improve antidepressant adherence in the management of depression? A targeted review and 10 clinical recommendations. Braz J Psychiatry. 2021 Mar-Apr;43(2):189-202. doi: 10.1590/1516-4446-2020-0935. PMID: 32491040; PMCID: PMC8023158.
